# The pedigree analysis and prenatal diagnosis of *Hong Kongαα* Thalassemia and the sequence analysis of *Hong Kongαα* Allele

**DOI:** 10.1002/mgg3.1285

**Published:** 2020-05-18

**Authors:** Wenjuan Wang, Haiqing Zheng, Dan Zeng, Linbin Jiang, Donglan Yu, Yuzhong Yang, Qiao Feng, Yang Xia, Chunjiang Zhu

**Affiliations:** ^1^ Department of Genetics Affiliated Hospital of Guilin Medical University Guilin People’s Republic of China; ^2^ Northwest Women’s and Children’s Hospital Xi’an People’s Republic of China; ^3^ College of Biotechnology Guilin Medical University Guilin People’s Republic of China; ^4^ Department of Pathology Affiliated Hospital of Guilin Medical University Guilin People’s Republic of China; ^5^ Department of Biochemistry and Molecuar Biology University of Texas Health Science Center at Houston Houston TX USA

**Keywords:** gene sequence, *Hong Kongαα* thalassemia, pedigree, prenatal diagnosis

## Abstract

**Background:**

Thalassemia is one of the most common monogenic hemolytic disorders in the world. *Hong Kongαα* (*HKαα*) thalassemia was initially found among the people of southern China. Because of the complexity of genetic changes in *HKαα* thalassemia, we lack a precise sequence analysis of the *HKαα* allele. Here we aim to detect the specific genotype and trace the law of inheritance of this rare genotype.

**Methods:**

We recruited an unprecedented huge pedigree containing 11 individuals carrying the *HKαα* thalassemia gene and 4 nongenetic‐related patients suffering from *HKαα* from south China. Regular hematological analysis and routine genetic screening were performed on the pedigree and two‐round nested PCR (polymerase chain reaction) for *HKαα* thalassemia were performed on each individual. The first‐generation gene sequencing was performed on six individuals, including four nongenetic‐related patients.

**Result:**

We found that five family members were positive for the *HKαα* allele. Patients Ⅱ‐2, Ⅲ‐1, and Ⅱ‐3 with only *HKαα/‐‐^SEA^* or *HKαα/‐α^4.2^* presented with α‐thalassemia minor trait. Ⅰ‐1, the carrier of both *HKαα/‐α^3.7^* and *β^41‐42^/β^N^*, showed a typical β‐thalassemia trait. Fetus with genotype *HKαα/‐α^4.2^* alone was not likely to suffer from any deleterious effects after birth. The whole sequence of *HKαα* allele revealed that *HKαα* alleles in the six patients shared a high similarity, implying that all *HKαα* alleles are likely from the same ancestor. Moreover, pedigree and sequencing analyses demonstrated that the *HKαα* allele contained ααα^anti4.2^ mutation, ‐α^3.7^ mutation, and a fragment from *α‐*hemoglobin gene; thus, the composition and formation of *HKαα* allele was revealed. Finally, the high similarity and composition of *HKαα* alleles implies that once HK*αα* formed, ααα^anti4.2^ and ‐α^3.7^ mutations tended to be a fusion gene and quite impossible to be inherited separately.

**Conclusion:**

The two‐round nested PCR is an effective method to detect *HKαα allele*. Besides, our study for the first time revealed the sequence of the *HKαα* allele, the evidence of the same ancestor with *HKαα* thalassemia and enriched the composition as well as the formation mechanism of *HKαα allele*, and immediately opened up novel potential diagnosis and prenatal counseling for *HKαα* thalassemia.

## Graphical abstract

A rarely huge pedigree of *HKαα* thalassemia is reported and an effective prenatal counseling is offered to this family. Furthermore, *αααanti4.2* detection assay is once again proposed to screen for *HKαα* allele in the patients positive for –*α3.7*mutation by our current studies. The relationship between genotypes and phenotypes of *HKαα* thalassemia was also analyzed here for clinicians to avoid the possible misdiagnosis. More importantly, our study for the first time reported the sequence of the *HKαα*allele, the evidence of the same ancestor with *HKαα* thalassemia and enriched the composition as well as the formation mechanism of *HKαα* allele，and immediately opened up novel potential diagnosis and prenatal counseling for *HKαα* thalassemia.

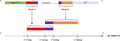

## 1 INTRODUCTION

Thalassemia, the most common monogenic disorder in the world (Weatherall & Clegg, 1996), is caused by genetic defects affecting hemoglobin gene expression. The reported carrier rate of α‐thalassemia in tropical and subtropical populations is about 1%, and has plateaued in some areas (Harteveld & Higgs, 2010). In Guangxi, China, the total heterozygous frequency of thalassemias and other hemoglobinopathies is 24.51%, in which α‐thalassemia accounts for 17.55% (Xiong et al., 2010). Mutations affecting the *alpha‐globin* gene lead to the pathogenic deficit of the alpha hemoglobin production in α‐thalassemia. Deletional mutations account for the majority of the α‐thalassemia cases, of which the SEA deletion (‐‐^SEA^), the rightward deletion (‐α^3.7^), and the leftward deletion (‐α^4.2^) contribute to up to 61.37%, 18.52%, and 6.80%, respectively, of the disease cases in Guilin, Guangxi, China (Tang et al., 2015). Thai type is also identified among the residents of Guilin, Guangxi, China. Additionally, the discovery of a more complicated genotype, *HongKongαα* (*HKαα*), which contains both *‐α^3.7^* and *ααα^anti4.2^* unequal crossover junctions (Wang, Chan, Chan, Ma, & Chong, 2005), further enriched the portfolios of the deletional types of α‐thalassemia. However, *HKαα* thalassemia is not as common as other deletional types of α‐thalassemia or point mutation types of β‐thalassemia, with a carrier rate of 0.07% (Shang et al., 2013) in the population of Guangxi, China. Because of this, only few studies on the diagnosis of *HKαα* thalassemia or its clinical manifestations are reported. For example, routine detections for deletional genotype of *α* thalassemia show the same results in *HKαα/αα* and ‐α^3.7^/αα; therefore, it is prone to be misdiagnosed. Besides, the hematological phenotype of an unborn fetus with *HKαα/‐‐^SEA^* is much milder than a baby with *‐α^3.7^/‐‐^SEA^* as the fetus does not present with HbH disease (Yao et al., 2015). However, it is difficult to distinguish these two types of genotypes by conventional clinical examination methods. Although the two‐round nested PCR is an effective method for *HKαα* thalassemia diagnosis (Wang et al., 2005), it is too complicated and uneconomical to perform in clinical laboratories. Thus, to identify accurate and convenient diagnosis and prenatal genetic counseling for *HKαα* thalassemia, we conducted a rarely huge pedigree containing 11 individuals and 4 nongenetic‐related patients suffering from *HKαα* thalassemia with multidisciplinary approaches. Here, we successfully identified the sequence of *HKαα* thalassemia and provide a novel potential diagnosis and prenatal counseling strategy for the disease.

## 2 MATERIALS AND METHODS

### 2.1 Ethical compliance

Informed consent forms were acquired from all of the participants. This study was approved by the ethics committee of Guilin Medical University, Guilin, Guangxi, PRC.

### 2.2 Patients

A pedigree containing 11 individuals (10 adults and 1 unborn fetus) carrying the *HKαα* thalassemia gene and 4 nongenetic‐related patients (S1, S2, S3, and S4) suffering from *HKαα* was recruited from different areas of Guangxi province, south China. The pedigree with 10 individuals shows no anemic phenotypes, except a patient with ‐α^3.7^/‐‐^SEA^. The five males and five females in the pedigree developed normally and showed neither skeletal deformity nor hepatosplenomegaly, and none of them ever took any treatment for thalassemia. Besides, the suspicious α2 junction fragment was detected in four nongenetic‐related individuals (S1, S2, S3, and S4). Thus, the samples of S1, S2, S3, and S4 were preserved for the subsequent gene sequence analysis.

### 2.3 Collection of samples

Peripheral blood of all of the participants (except the unborn fetus) was collected in EDTA‐containing tubes for hematological phenotype analysis. Genomic DNA was extracted to determine their thalassemia genotypes. A quantity of 8 ml of amniotic fluid was collected from the proband's spouse (Ⅱ‐1) for the extraction of the fetal genomic DNA to determine the genotype of the unborn fetus. We failed to obtain the sample from the spouse of Ⅱ‐3 because of some insurmountable difficulties. A magnetic bead adsorption/automatic nucleic acid extraction method (Zhishan Biotechnology) was utilized for DNA extraction.

### 2.4 Hematological analysis

Whole blood cell counts were performed on all the participants, except Ⅲ‐2, with an automated cell counter (Model Sysmex F‐820; Sysmex Co Ltd, Kobe, Japan), and hemoglobin electrophoresis tests were conducted on a capillary electrophoresis (CE) device (Capillarys, Sebia, Montpellier, France).

### 2.5 Gene analysis

#### 2.5.1 Detection of deletional genotype of α Thalassemia

**Figure 1:**
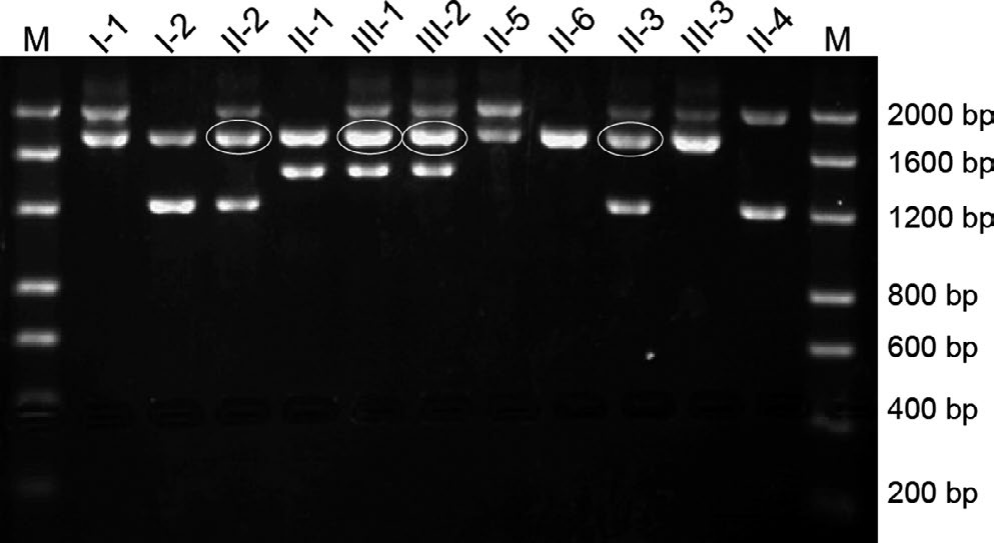
S‐tube multiplex PCR for four common deletional types of α‐thalassemia. The single‐tube multiplex PCR for four common deletional types of α‐thalassemia indicated the anomalous PCR amplification fragments in II‐2, II‐3, III‐1, and III‐2， which are marked by ovals

The single‐tube multiplex polymerase chain reaction (PCR) (Tan, Quah, Low, & Chong, 2001) for four Chinese common deletional α‐thalassemia types (‐*α^3.7^*,‐*α^4.2^*,‐‐*^SEA^*, and ‐‐*^THAI^*) was performed on the pedigree to determine the deletional genotype of α‐thalassemia and the suspicious *HKαα* allele (Figure 1).

#### 2.5.2 ααα^anti4.2^ allele detection

**Figure 2:**
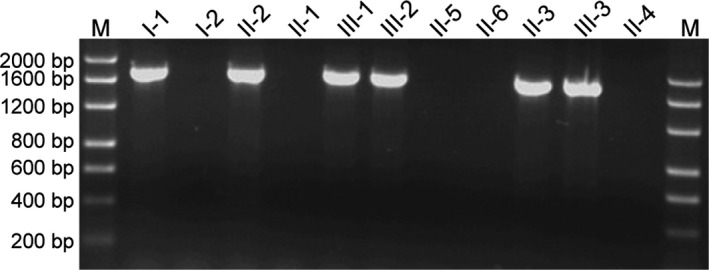
*ααα^anti4.2^* junction fragment detection. The *ααα^anti4.2^* junction fragment was detected in I‐1, II‐2, III‐1, III‐2, II‐3, and III‐3

Polymerase chain reaction for the detection of the *ααα^anti4.2^* allele was performed to determine the probability of the presence of *HKαα* allele and to identify the genotype of *HKαα/αα*, *HKαα/α^3.7^*, and ‐*α^3.7^/αα* as all of them show similar results in conventional detection methods of deletional genotypes of α‐thalassemia. The primer sequences and the PCR reaction were performed as described by Wen et al. (2003) (Figure 2).

#### 2.5.3 Two‐round nested PCR on the pedigree

**Figure 3:**
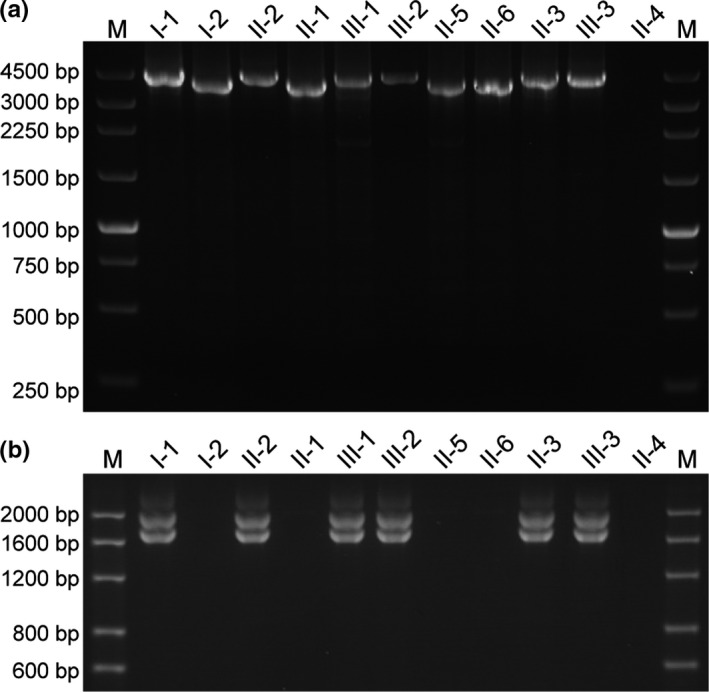
Two‐round nested PCR for *HKαα* allele on the pedigree. a: In round 1 of two‐round nested PCR, I‐1, II‐2, III‐1, III‐2, II‐3, and III‐3 who were positive for *HKαα* allele yielding the PCR amplification fragments of 4.5 kb. b: In round 2 of two‐round nested PCR，I‐1, II‐2, III‐1, III‐2, II‐3, and III‐3 were positive for both *‐α^3.7^* and *ααα^anti4.2^* mutations, which proved that they were the carriers of *HKαα* thalassemia

The two‐round nested PCR on pedigree members was employed to determine the presence of the *HKαα* allele. The primer sequences and the reaction conditions were same as that described in previous articles (Wang et al., 2005; Tan et al., 2001; Wen et al., 2003; Figure 3).

#### 2.5.4 Detection of point mutation of *α‐* and *β‐*thalassemia

**Figure 4:**
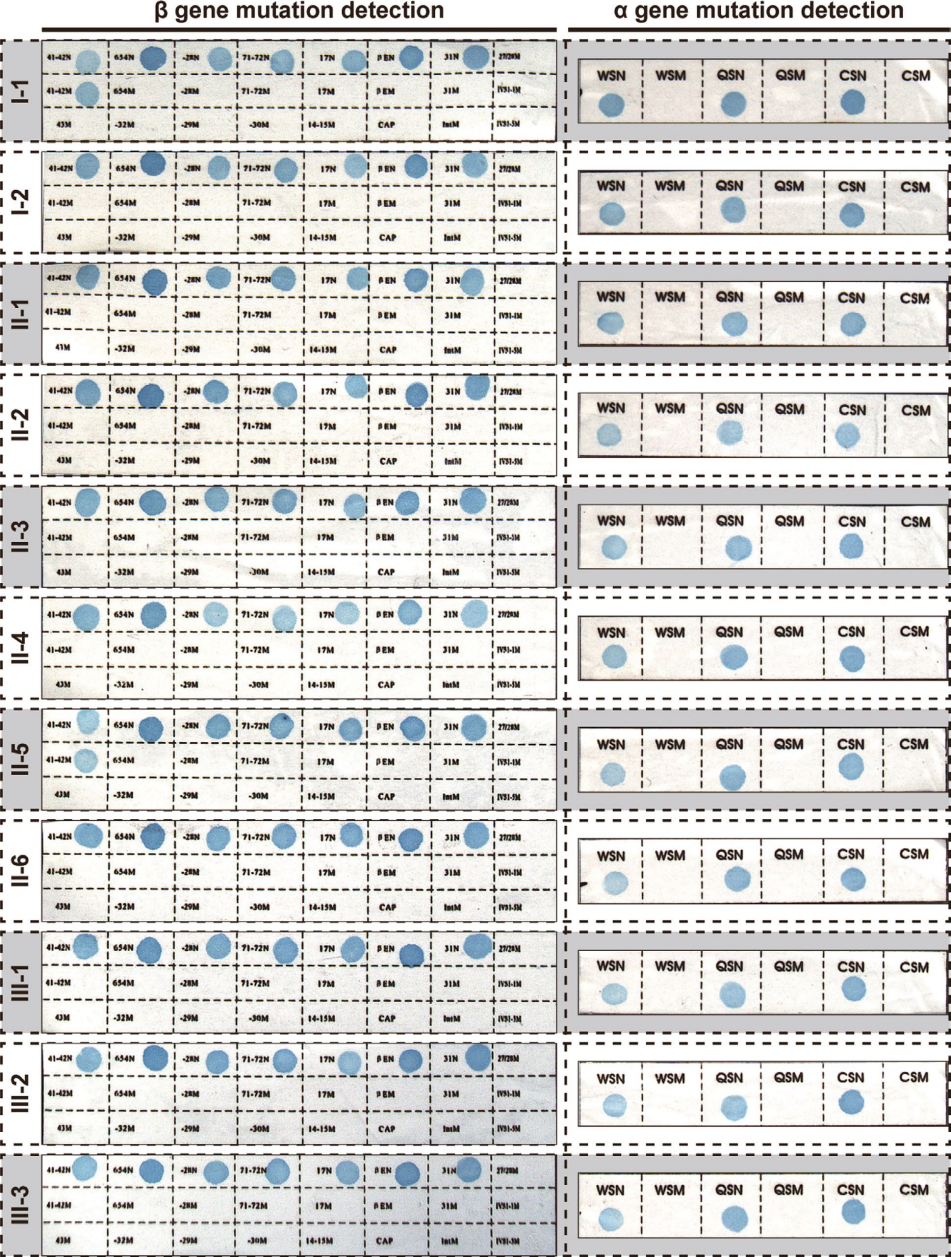
Results of the reverse dot blot for β‐ and α‐gene mutation detection. The reverse dot blot for β‐gene mutation detection showed that I‐1 and II‐5 were the carriers of β41‐42 mutation, while I‐2, II‐1, II‐2, II‐3, II‐4, II‐6, III‐1, III‐2, and III‐3 are negative for β‐thalassemia. All the participants are negative in nondeletional α‐gene mutation

A reverse dot blot (RDB) analysis was performed on the pedigree to detect nondeletional mutations of *α‐* and *β‐*thalassemias (Figure 4).

#### 2.5.5 Two‐round nested PCR on unrelated individuals

We redesigned the primer for the first round of the two‐round nested PCR as follows: ZW‐F：5′‐CTCGGTAGCCGTTCCTCCTGC‐3′, ZW‐R 5′‐AAGTCTGGGAATAAAACTCGGGA‐3′.

**Figure 5:**
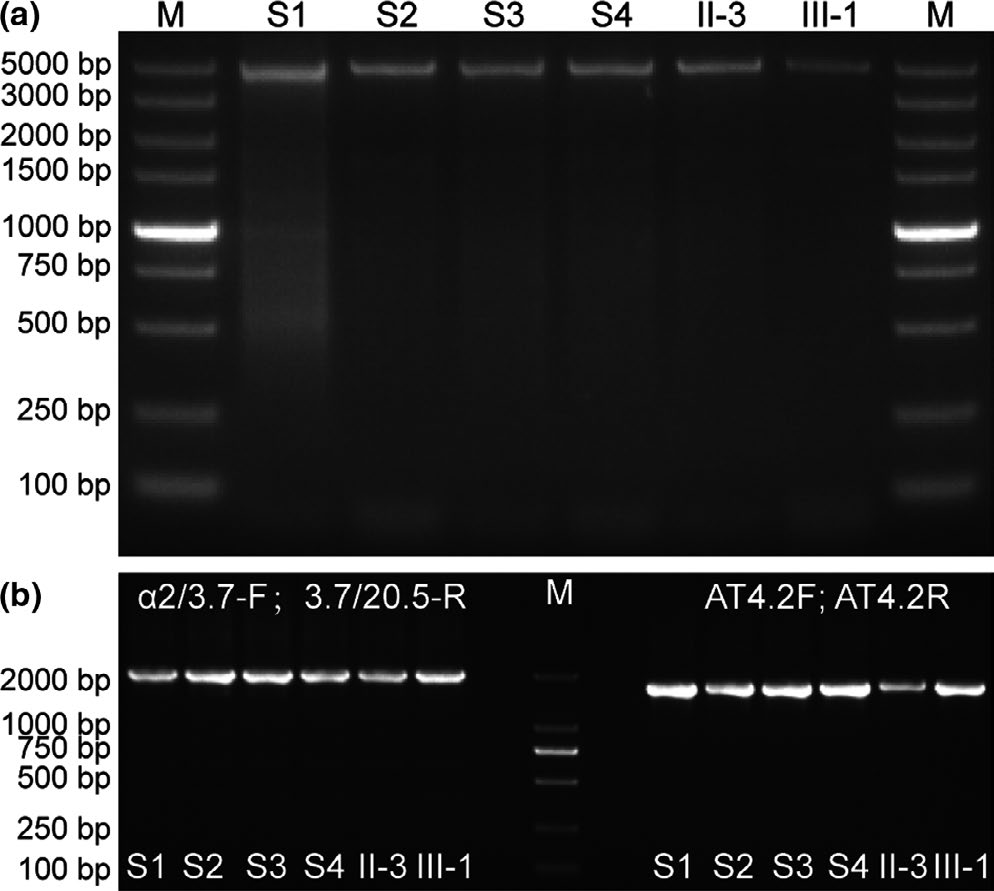
Two‐round nested PCR for *HKαα* allele for six individuals. a: In round 1 of two‐round nested PCR，S1, S2, S3, S4, II‐3, and III‐1 who were positive for *HKαα* allele yielding the PCR amplification fragments of 4.5 kb. b: In round 2 of two‐round nested PCR， S1, S2, S3, S4, II‐3, and III‐1 were positive for both *‐α^3.7^* and *ααα^anti4.2^* mutations, which proved that they were the carriers of *HKαα* thalassemia

The reaction procedure was optimized as follows: initial denaturation at 95°C for 5 min was followed by five cycles：94°C for 30 s, 70°C for 30 s, decreased 1°C for every cycle, 72°C for 4 min, and then 35 cycles of 94°C for 30 s, 65°C for 30 s, 72°C for 4 min, and a final 72°C for 5 min. Primers for round 2 were same as that used in previous articles (Tan et al., 2001; Wen et al., 2003). The reaction procedure of round 2 was also optimized as follows: initial denaturation at 95°C for 5 min was followed by five cycles： 94°C for 30 s, 64°C for 30 s, decreased 1°C for every cycle, 72°C for 4 min, and then, 35 cycles of 94°C for 30 s, 59°C for 30 s, 72°C for 4 min, and a final 72°C for 5 min (Figure 5).

### 2.6 Sequencing the genomic DNA

DNA samples of S1, S2, S3, S4, Ⅱ‐3, and Ⅲ‐1 were sequenced using first‐generation gene sequencing on ABI (3730XL) to explain the sequence, the structure, the origin, and to explore the probability of simplifying the diagnosis of *HKαα* thalassemia allele.

### 2.7 Explanation of HKαα thalassemia allele

The *HKαα* thalassemia alleles of all six sequenced samples were compared with each other. The *HKαα* thalassemia allele was separated into three sections: the ‐α^3.7^allele, the ααα^anti4.2^ allele, and a special allele.

## 3 RESULTS

### 3.1 Recruitment of an unprecedented huge pedigree containing 11 individuals carrying the HKαα thalassemia allele

The proband's wife (Ⅱ‐1) was diagnosed as the carrier of thalassemia gene ‐α^4.2^/αα when pregnant. Next, routine thalassemia screening tests and genetic tests were given to the proband (Ⅱ‐2). Surprisingly, a suspicious α2 junction fragment was discovered besides the *‐α^3.7^*and *‐‐^SEA^* junction fragments. This unexpected result led us to suspect the probable presence of the *HKαα* allele as reported. To investigate this possibility, to explore the law of the inheritance of this genotype, as well as to provide accurate and effective prenatal counseling regarding the proband's unborn fetus (Ⅲ‐2), she and 10 other family members were recruited in this research.

### 3.2 Genotyping the pedigree containing 11 individuals carrying HKαα thalassemia gene

The five males (Ⅰ‐1, Ⅱ‐2, Ⅱ‐5, Ⅲ‐1, and Ⅲ‐3), five females (Ⅰ‐2, Ⅱ‐1, Ⅱ‐3, Ⅱ‐4, and Ⅱ‐6), and the proband's unborn fetus (Ⅲ‐2) involved in this research were genetically related. First, we conducted the RDB analysis and found that Ⅰ‐1 and Ⅱ‐5 were carriers of the *CD 41–42(‐CTTT)* mutation (Figure 4). Moreover, none of the individuals in this pedigree carried a nondeletional α‐thalassemia gene mutation (Figure 4).

To detect the four common deletional types of α‐thalassemia, we used single‐tube multiplex PCR analyses. We found the presence of an anomalous fragment in Ⅱ‐2 and Ⅱ‐3, who were also positive for *‐‐^SEA^* and ‐*α^3.7^* alleles, as well as in Ⅲ‐1 and Ⅲ‐2 who were also positive for ‐*α^3.7^* and *‐α^4.2^* alleles (Figure 1). Then, the *ααα^anti4.2^* junction fragment detection assay demonstrated that Ⅰ‐1, Ⅱ‐2, Ⅲ‐1, Ⅲ‐2, Ⅱ‐3, and Ⅲ‐3 were positive for the *ααα^anti4.2^* allele (Figure 2), which implied that these individuals were carriers of *HKαα* thalassemia gene. The subsequent two‐round nested PCR proved that Ⅰ‐1, Ⅱ‐2, Ⅲ‐1, Ⅲ‐2, Ⅱ‐3, and Ⅲ‐3 were carriers of the *HKαα* allele. Taken together, the genotypes of the patients are listed as follows: Ⅰ‐1 positive for both *HKαα/‐α^3.7^* and*β^41‐42^/β^N^*, Ⅰ‐2 with *‐‐^SEA^/αα*, Ⅱ‐1 with *‐α^4.2^/αα*, Ⅱ‐2 with *HKαα/‐‐^SEA^*, Ⅱ‐3 with *HKαα/‐‐^SEA^*, Ⅱ‐4 with*‐α^3.7^/‐‐^SEA^*, Ⅱ‐5 with both‐*α^3.7^/αα* and *β^41‐42^/β^N^*, Ⅱ‐6 with normal *αα/αα*, Ⅲ‐1 with *HKαα/‐α^4.2^*, Ⅲ‐2 with *HKαα/‐α^4.2^*, and Ⅲ‐3 with an uncertain genotype.

### 3.3 Correlation of genotypes to phenotypes in the pedigree of 11 Individuals with *HKαα thalassemia*

**Figure 6:**
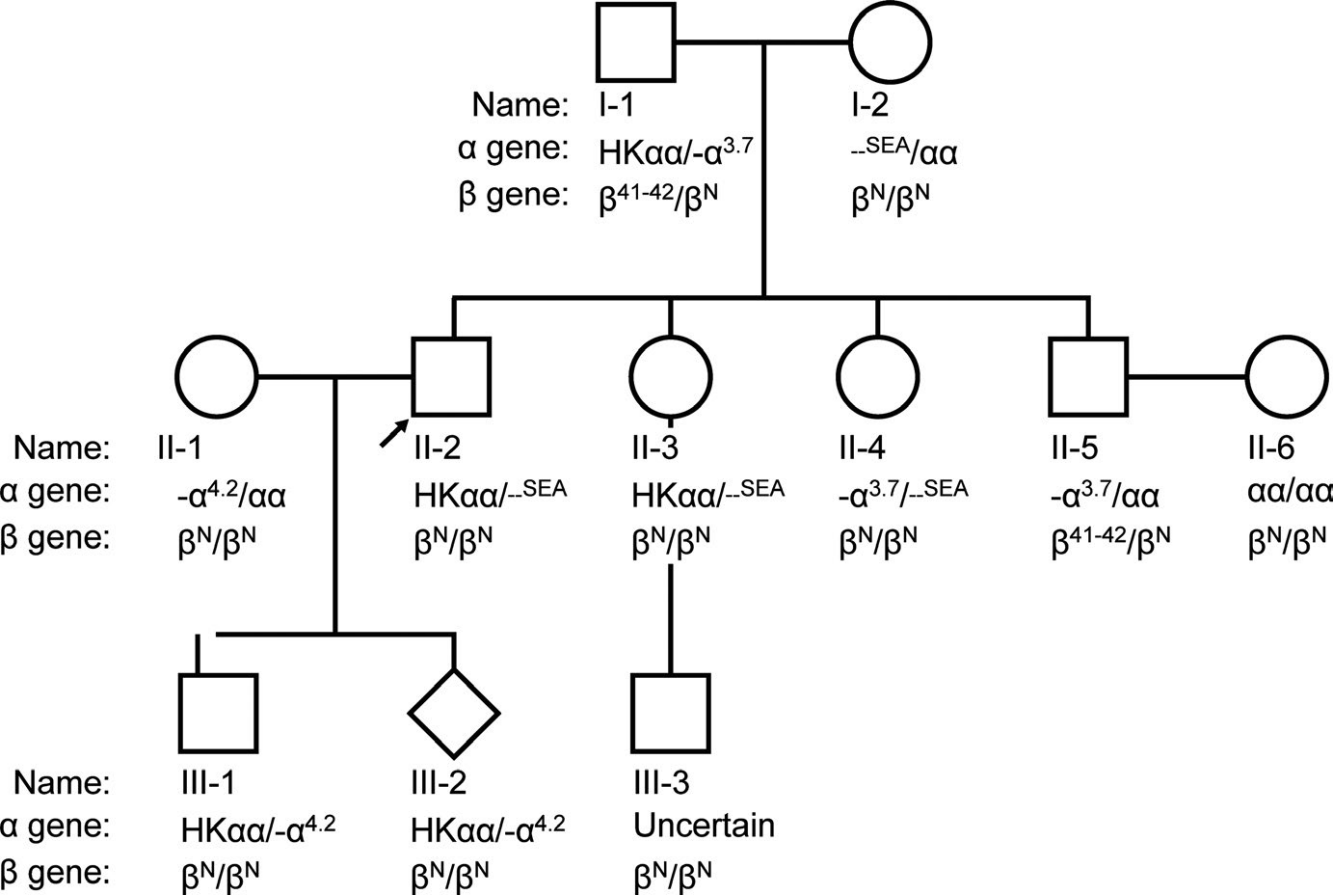
Family Tree. The proband is marked with the arrow. The genotype of III‐3 will not be determined until the spouse of II‐3 is genotyped

Patients Ⅱ‐2, Ⅲ‐1, and Ⅱ‐3 with only *HKαα/‐‐^SEA^* or *HKαα/‐α^4.2^* presented α‐thalassemia trait. Ⅰ‐1, who was the carrier of both *HKαα/‐α^3.7^* and *β^41‐42^/β^N^*, showed a typical β‐thalassemia trait characterized by increased HbA2 (6.4%) and microcytic hypochromic anemia with mild reduction in mean cell volume (MCV), mean cell hemoglobin (MCH), and mean corpuscular hemoglobin concentration (MCHC). Besides, the genotype of the unborn fetus was *HKαα/‐α^4.2^*, and its phenotype was theoretically identical to that of Ⅲ‐1 who shares the same thalassemia genotype with the unborn fetus (Table 1, Figure 6).

**Table 1:** Patient Information in the *HKαα* pedigree

Samples	Gender	Age (year)	Phenotype						Genotype	
			RBC (×10^12^/L)	HGB (g/L)	MCV (fl)	MCH (pg)	MCHC (g/L)	HbA2 (%)	*α*	*β*
I−1	Male	64	5.41	104	64.9	19.2	296	6.4	HKαα/‐α^3.7^	β^41−42^/β^N^
I−2	Female	63	5.38	118	73.4	21.9	299	2.3	‐‐‐‐^SEA^/αα	β^N^/β^N^
II−1	Female	34	4.03	104	79.7	25.8	324	2.6	‐α^4.2^/αα	β^N^/β^N^
II−2	Male	35	6.98	139	64.6	19.9	308	2.5	HKαα/‐‐^SEA^	β^N^/β^N^
II−3	Female	30	5.85	123	70	21	296	2.5	HKαα/‐‐^SEA^	β^N^/β^N^
II−4	Female	41	5.38	90	58.6	16.7	286	1.3	‐α^3.7^/‐‐^SEA^	β^N^/β^N^
II−5	Male	38	7.12	134	63.3	18.8	297	6.4	‐α^3.7^/αα	β^41−42^/β^N^
II−6	Female	36	4.69	145	93.6	30.9	330	2.9	αα/αα	β^N^/β^N^
III−1	Male	9	5.12	126	74.2	24.6	332	2.9	HKαα/‐α^4.2^	β^N^/β^N^
III−2	Unknown	0	Unknown	Unknown	Unknown	Unknown	Unknown	Unknown	HKαα/‐α^4.2^	β^N^/β^N^
III−3	Male	3	5.58	133	74.4	23.8	320	3.2	HKαα/?	β^N^/β^N^

Note

Table presents the clinical phenotypes and genotypes of all individuals of the family carrying *HKαα* gene. Ⅰ‐1, Ⅱ‐2, Ⅱ‐3, Ⅲ‐1, Ⅲ‐2, and Ⅲ‐3 were all positive for *HKαα* mutation. These carriers were diagnosed as the patients with microcytic hypochromic anemia. Ⅰ‐1 was identified to be the carrier of both *HKαα/‐α^3.7^* and *β^41‐42^/β^N^* with increased HbA2(6.4%) and decreased HGB, MCV, MCH, and MCHC.

### 3.4 HKαα gene sequence

**Figure 7:**
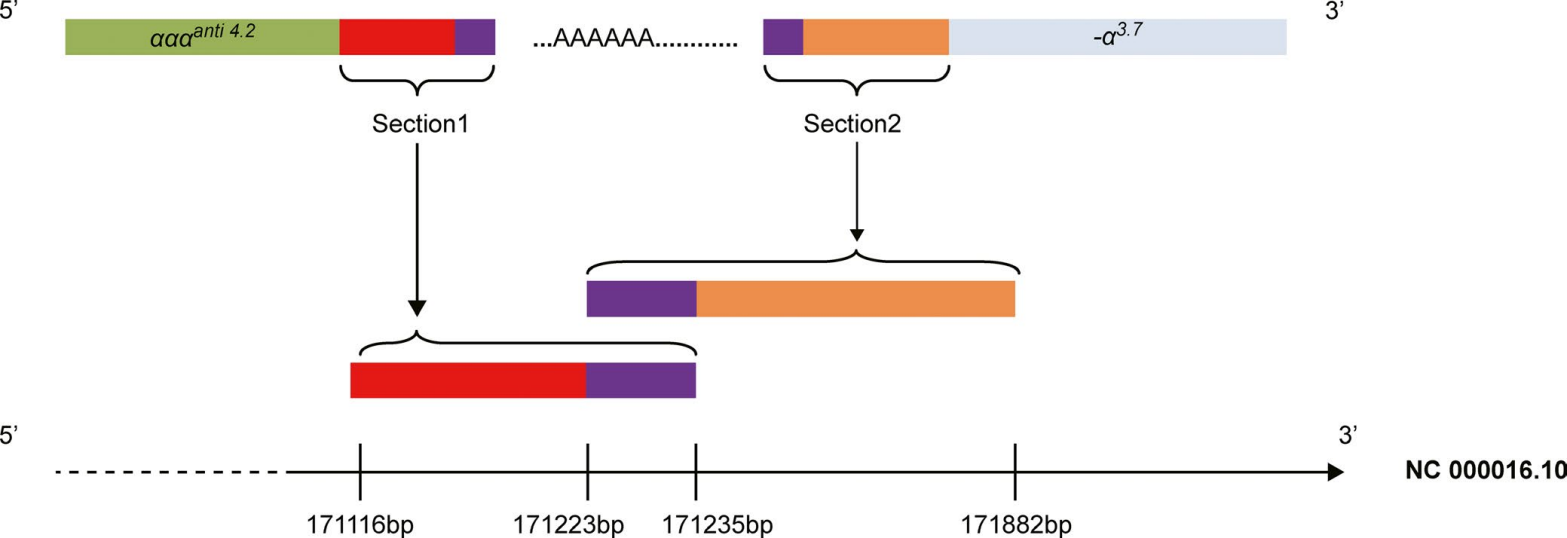
Structure Of HKαα Allele. The green block stands for *ααα^anti4.2^* allele. Section 1 stands for upstream region which shares the same sequence with that on alpha globin gene ranging from 171,116 to 171,235. Section 2 stands for the downstream region which is consistent with that on alpha globin gene ranging from 171,223 to 171,882, (NCBI Reference Sequence: Ng_0000016.10). The blue block stands for *‐α^3.7^* allele. The purple block stands for the same sequence which section 1 and section 2 share

The two‐round nested PCR proved Ⅱ‐3，Ⅲ‐1，S1，S2，S3，and S4 are carriers of *HKαα* (Figure 5). Sequence analyses were performed. *HKαα* gene sequence data were provided in supplemental data. Specifically, sequence analysis showed that the sequences of the *HKαα* allele of Ⅱ‐3，Ⅲ‐1，S1，S2，S3，and S4 were highly similar. We cut this sequence into three segments: *‐α^3.7^* allele, *ααα^anti4.2^* allele, and the special allele about 802 base pairs, except the sequences we failed in detecting. The comparison between the gene sequence of the whole human genome and the special allele of 802 base pairs indicated that this 802 base pairs could be divided into three regions: the upstream region shared the same sequence with that on *alpha globin* gene ranging from 171,116 to 171,235, the downstream region was consistent with that on *alpha globin* gene ranging from 171,223 to 171,882, and astride them is the sequence we failed in detecting (NCBI Reference Sequence: Ng_0000016.10) (Figure 7).

## 4 DISCUSSION

The human hemoglobin is a tetramer protein comprising a pair of α globin chains and a pair of β globin chains, encoded by the α globin genes located on the chromosome 16p13.3 and β globin genes located on the chromosome 11p15.3, respectively. α‐thalassemia, in which the *‐α^3.7^*, ‐*α^4.2^*, and ‐‐^SEA^ deletion are commonly detected in south China, mainly arises from the deletion of *α‐globin* genes. The α‐globin gene sequence on the α‐gene cluster can be described as: 5’‐ζ‐ψζ1‐ψα2‐ψα1‐α2‐α1‐θ‐3’, in which both α1 and α2 genes including *X, Y,* and *Z hemoglobin boxes* are embedded within a homologous region. The *–α^3.7^* gene type is caused by a reciprocal recombination between Z segments, and thereby contributes to a functional α gene and an α‐triplication. Similarly, the recombination between X boxes can lead to the *‐α^4.2^* gene type (Harteveld & Higgs, 2010). The *HKαα* allele contains an α1‐α2 fusion gene (–α^3.7^) and an intact α2 gene (Wu et al., 2015a), Although some mechanisms have been proposed to explain the origination of the *HK* allele (Wang et al., 2005), there is no certain conclusion for it yet.

As confirmed in the two‐round nested PCR, Ⅰ‐1 was a carrier of *HKαα* thalassemia. Thus, the negative result revealed by four deletional α‐thalassemia detection assays could be explained by the possibility that the α2 gene band serving as the reminder of *HKαα* allele was concealed by the normal α band. Hence, theoretically, there could be three possible genotypes of Ⅰ‐1 including *HKαα/αα*, *HKαα/‐α^3.7^*, and *HKαα/HKαα*. As shown in Figure 6, Ⅱ‐2, Ⅱ‐3, Ⅲ‐1, Ⅲ‐2, and Ⅲ‐3 were all positive for *HKαα* allele. Although this genotype includes both *ααα^anti4.2^* and *‐α^3.7^*, it is quite impossible that *ααα^anti4.2^* and *‐α^3.7^* are inherited separately. Furthermore, the sequencing results showed that the *HKαα* allele in S1, S2, S3, S4, Ⅱ‐3, and Ⅲ‐1 are quite consistent with each other, indicating that *HKαα* allele is highly stable as a fusion gene. As Ⅱ‐4 is a heterozygote of *‐α^3.7^* deletion and *–^SEA^* deletion, the genotype of Ⅰ‐2 is *‐‐^SEA^/αα*. As the *ααα^anti4.2^* and *‐α^3.7^* mutations in *HKαα allele* are not possible to inherit separately, it could be deduced that Ⅰ‐1 is the carrier of *‐α^3.7^* allele. Therefore, the genotype of Ⅰ‐1 could be *HKαα/‐α^3.7^*. Similarly, the genotype of III‐3 relies on the determination of II‐3’s spouse's genotype. Our future research will focus on distinguishing *HKαα/αα*, *HKαα/‐α^3.7^*, and *HKαα/HKαα*.

The two‐round nested PCR is a reliable method to detect the presence of *HKαα allele*, and the pedigree analysis should be employed to determine the precise genotype. As the carrier rate of *HKαα* allele in Guangxi is 0.07% (Shang et al., 2013), and it is prone to be misdiagnosed as *‐α^3.7^*/*αα* mutation, the actual carrier rate of *HKαα* allele in Guangxi may be much higher. We suggest more attention should be paid to the diagnosis of HKαα thalassemia. Accordingly, *ααα^anti4.2^* allele detection assay should be performed once a patient is identified positive for –*α^3.7^* deletional type. Furthermore, to identify whether the *ααα^anti4.2^* and ‐*α^3.7^* are present on the same or two different α genes, the two‐round nested PCR should be performed if the presence of *ααα^anti4.2^* allele is confirmed.

β‐Thalassemia is characterized by microcytic hypochromic anemia as well as increased HbA2. Either blood routine examination or hemoglobin electrophoresis is hitherto the main method for its screening. Thus, the patients with both *HKαα* thalassemia and β‐thalassemia are prone to be diagnosed as β‐thalassemia only by routine tests. Typical β‐thalassemia minor may interfere with the diagnosis of *HKαα* thalassemia. For example, in the pedigree shown in Table 1, Ⅰ‐1 is positive for both *HKαα/‐α^3.7^* and *β^41‐42^/β^N^*. Its routine blood test revealed microcytic hypochromic anemia with MCV 64.9 fl, MCH 19.2 pg, MCHC 296 g/L, and an increase in HbA2(6.4%). However, the possibility of *HKαα* thalassemia would not be usually considered based on these testing results.

Since there is no change in the abundance of the α gene in *HKαα* allele (Shang et al., 2013; Wang et al., 2005; Wu et al., 2015b). The clinical presentation of Ⅱ‐3 and Ⅱ‐2 is much milder than that of Ⅱ‐4 as shown in Table 1. Ⅱ‐3, who was positive for *HKαα/‐‐^SEA^*, is associated with minor thalassemia symptoms with red blood cell 5.85 × 10^12^, HGB 123 g/L, MCV 70 fl, MCH 21 pg, and MCHC 296 g/L. Similarly, Ⅱ‐2, with the same genotype as Ⅱ‐3, presents minor thalassemia features with RBC 6.98 × 10^12^, HGB 139 g/L, MCV 64.6 fl, MCH 19.9 pg, and MCHC 308 g/L. However, Ⅱ‐4, who is positive for *–α^3.7^/‐‐^SEA^*, shows the symptoms of HbH disease with RBC 5.38 × 10^12^, HGB 90 g/L, MCV 58.6 fl, MCH 16.7 pg, and MCHC 286 g/L. Finally, the unborn fetus is unlikely to suffer from any deleterious effects though its genotype was *HKαα/‐α^4.2^*, which is identical to that of Ⅲ‐1 (Table 1). In addition, based on the fact that there is no change in the abundance of α gene in HK*αα* allele (Wu et al., 2015c), the clinic symptoms of the unborn fetus could also be predicted to be similar to those of individuals with *‐α^4.2^/αα*.

The sequencing results confirmed that *HKαα* allele in Ⅱ‐3，Ⅲ‐1，S1，S2，S3，and S4 are quite similar, which strongly indicates that individuals with *HKαα* thalassemia inherit the allele from the same ancestor since Ⅲ‐1，S1，S2，S3，and S4 were not genetically related. Although this opinion was also proposed by Shang et al. (2013), we are the first to describe the sequence of *HKαα* allele and to offer the convincing evidence.

As is mentioned above, the sequenced *HKαα* allele can be divided into three regions: *ααα^anti4.2^* allele, *‐α^3.7^* allele, and between them is the upstream region which shares the same sequence with that on alpha globin gene ranging from 171,116 to 171,235, the downstream region which is consistent with that on alpha globin gene ranging from 171,223 to 171,882 (NCBI Reference Sequence: Ng_0000016.10) and astride them are the sequences we failed in detecting. This implies a complicated formation mechanism of *HKαα* allele and points out the need for further investigation.

Although the two‐round nested PCR is an effective method to identify *HKαα* allele, it is time‐consuming and cannot be commonly used in clinical diagnosis. As we have already revealed the sequence of *HKαα* precisely*,* we will continue to analyze its sequence and try to discover unique base pairs as its marker, so that we can simplify the detection of *HKαα* thalassemia.

In conclusion, a rarely huge pedigree of *HKαα* thalassemia is reported and an effective prenatal counseling is offered to this family. Furthermore, *αααanti4.2* detection assay is once again proposed to screen for *HKαα* allele in the patients positive for *–α3.7* mutation by our current studies. The relationship between genotypes and phenotypes of *HKαα* thalassemia was also analyzed here for clinicians to avoid the possible misdiagnosis. More importantly, our study for the first time repored the sequence of the *HKαα* allele, the evidence of the same ancestor with *HKαα* thalassemia and enriched the composition, as well as the formation mechanism of *HKαα allele*. These findings have significantly advanced our understanding of *HKαα* thalassemia and immediately provided a novel potential diagnosis and prenatal counseling approach for *HKαα* thalassemia.

## CONTRIBUTORSHIP STATEMENT

Chunjiang Zhu, Haiqing Zheng, Dan Zeng, Donglan Yu, and Qiao Feng collected the samples. Wenjuan Wang and Chunjiang Zhu designed the research, conducted the research, and analyzed the research results. Wenjuan Wang wrote the paper. Chunjiang Zhu, Wenjuan Wang, Yang Xia, and Yuzhong Yang revised the paper. Linbin Jiang provided the technical support.

## CONFLICT OF INTEREST

The authors declare no competing financial interests.

## Supporting information

Supplementary Material

## Data Availability

All data involved in this manuscript are available.
