# Supplementary material for: The pedigree analysis and prenatal diagnosis of Hong Kongαα Thalassemia and the sequence analysis of Hong Kongαα Allele
Source: Mol Genet Genomic Med. 2020 May 18;8(7):e1285. doi: 10.1002/mgg3.1285 (PMC7336738; doi:10.1002/mgg3.1285)
Supplement: Supplementary file 1 — Supplementary Material [file MGG3-8-e1285-s001.pdf]

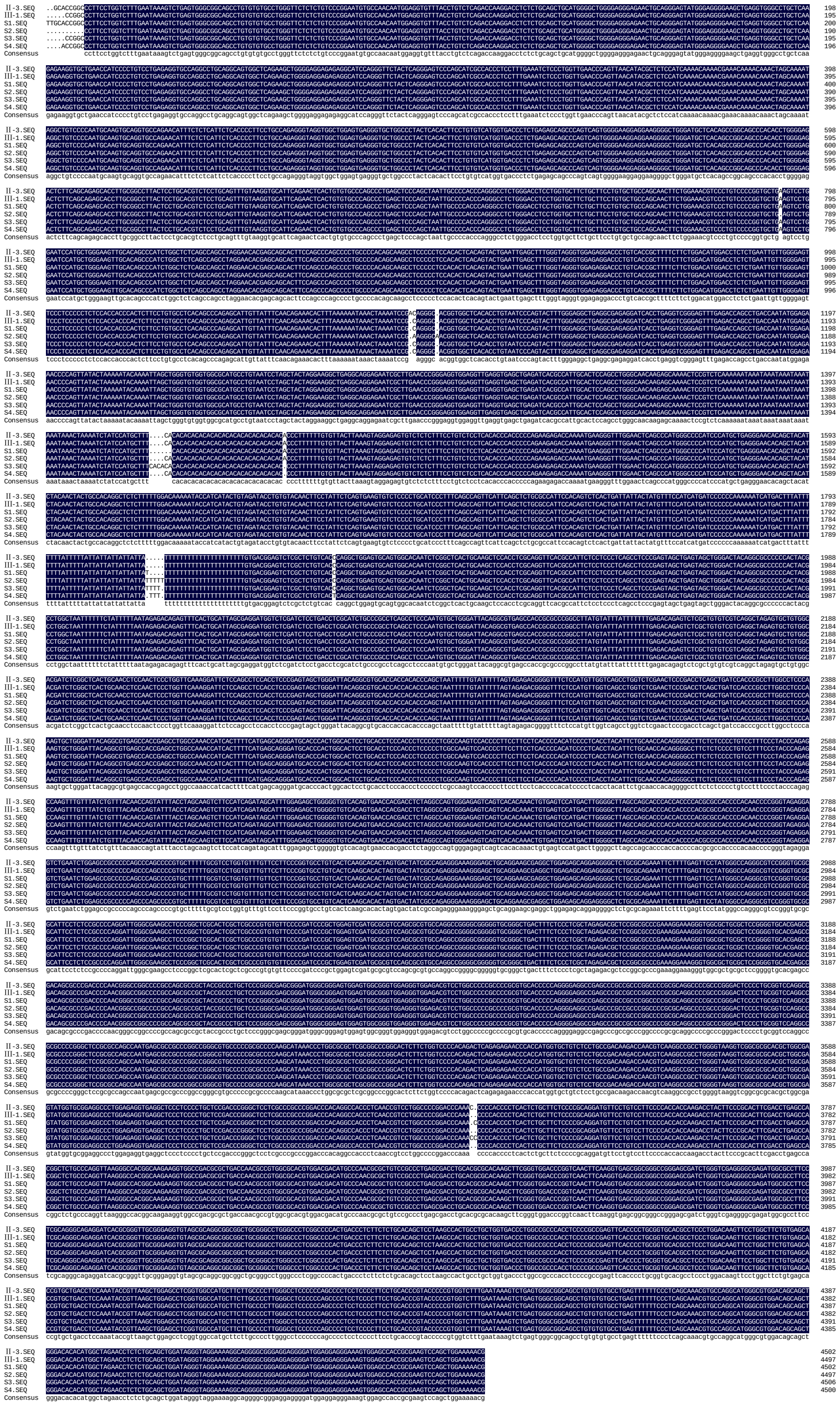

Alike sequences are labelled with blue. The ellipses stand for the sequence we failed in detecting or which are different from each other.
